# Assessment of fracture resistance in endodontically treated premolars restored using various bulk-fill composites

**DOI:** 10.6026/973206300222553

**Published:** 2026-04-30

**Authors:** Parthivi Singh, Yogita D. Deshpande, Kalpana S. Rai, Prathmesh Rai, Pooja Sinha, Manika Gautam

**Affiliations:** 1 Department of Conservative dentistry and Endodontics, Peoples dental Academy, Peoples University, Bhopal, Madhya Pradesh, India; 2 Department of Conservative dentistry and Endodontics, Guru Gobind Singh College of Dental Science and Research Centre, Burhanpur, Madhya Pradesh, India

**Keywords:** Fracture resistance, endodontically treated teeth, bulk fill composite, premolars, restorative dentistry

## Abstract

Endodontically treated premolars are structurally compromised and highly susceptible to fracture, necessitating restorative materials that can effectively reinforce tooth strength. Therefore, it is of interest to evaluate and compare the fracture resistance of endodontically treated maxillary premolars restored with three bulk-fill composites and a conventional incremental composite. Eighty premolars were endodontically treated, prepared with standardized MOD cavities and allocated into four groups (n=20): Filtek One Bulk Fill, Tetric EvoCeram Bulk Fill, SonicFill 3 and Filtek Z250. Fracture resistance was tested using a universal testing machine and analyzed with one-way ANOVA and chi-square tests. SonicFill 3 demonstrated the highest fracture resistance, followed by other bulk-fill composites, while the conventional composite showed the lowest values, indicating superior reinforcing potential of bulk-fill materials.

## Background:

The opportunity of endodontically treated teeth is one of the most uncommon restorative challenges in clinical practice because of the impaired structural integrity. The progressive loss of tooth structure caused by carious participation, access cavity preparation, root canal instrumentation and irrigation guidelines causes massive losses in the mechanical strength of the remaining tooth structure [1]. It is widely recorded that teeth whose endodontic treated have a very high susceptibility to fracture when compared to vital teeth and that premolar teeth are especially susceptible because their anatomical structure has high cusps and relatively thin remaining walls after access of endodontic treatment [2]. Endodontically treated premolars have a multifactorial structural weakening. In addition to the fact that coronal tooth structure is directly lost, alterations in the dentin composition after endodontic treatment make the issue. Dehydration of dentin, loss of the proprioceptive system offered by the vital pulp and also changes in the crosslinking of collagen that occurs in dentin all lead to the fact that the fracture threshold of such teeth decreases [3]. Premolars since they are exposed to both the axial and lateral occlusal forces during functioning, they are at high risk of catastrophic cusp fractures which can make the tooth unrestorable [4]. Conventionally, post-and-core systems and subsequent full-coverage crowns have been used to restore endodontically treated premolars that have been extensively corrosion-debrided. Nevertheless, the method requires further ablation of sound tooth structure, is time-consuming and more expensive [5]. Direct adhesive restorations have emerged as a valid and more conservative option to restore endodontically treated posterior anterior teeth with moderate tissue loss with the advances in adhesive dentistry and the composite resin technology [6]. Direct composite restorations have the benefit of preservation of remaining tooth structure as well as adequate mechanical reinforcement by providing adhesive bonding [7]. However, traditional composite resins have structural constraints that are inherent in placing them in very large posterior cavities. The process is technique-sensitive and time consuming due to the need of gradual insertion into the layers of no more than 2 mm whereby the polymerization reduction influences the final product. Every increment provides the potential of incorporating nothingness and contamination amid layers and can undermine the mechanical strength of the restoration [8]. Besides, stresses caused by polymerization shrinkage at every bonded interface can cause gaps, cuspal deflection and eventual marginal breakdown [9].

A solution to these weaknesses was to create bulk-fill composite resins that could be placed in 4 to 5 mm depth increments without reducing the level of cure or creating excessive polymerization shrinkage stress. Such materials include photoinitiator systems that have been modified, stress-relieving monomers and the specifically designed filler technologies that allow sufficient light transmission at higher thicknesses, without losing reasonable mechanical properties [10]. There are a number of types of bulk fill composites, such as those with flowable base materials which need capping by a traditional composite, those with paste- like consistency that can be shaped and can be used as full body restoratives and sonic-activated systems that become less viscous during the placement to allow better adaptation [11]. The mechanical behaviour of bulk-fill composites in the endodontically treated teeth has been of great research interest. A number of studies have also tested the fracture resistance of endodontically treated molars that were restored using different bulk-fill materials with mostly positive results [12]. Nevertheless, related data with a limited scope to endodontically treated premolars with a different set of anatomic and biomechanical concerns are still relatively scarce [13]. Also, the mechanical properties of more recent generation bulk-fill composites, including SonicFill 3 with its exclusive sonic-activation technology, have not been compared extensively with those of other bulk-fill systems in the context of endodontically treated premolars specifically [14]. Fracture resistance of a restored tooth not only depends on the property of the material but also depends on the quality of the adhesive interface; cavity configuration; and stress distribution pattern in the remaining tooth structure [15]. The potential of the restorative material to evenly distribute the occlusal stresses and strengthen the weakened cuspal walls is the highest consideration in the long-term survival of the endodontically treated premolars [16]. In addition the clinical significance of fracture patterns; fractures that are restorable or catastrophic, is as important as absolute values of fracture resistance in establishing the clinical prognosis [17]. Therefore, it is of interest to compare and contrast fracture resistance of endodontically-treated maxillary premolars restored using three bulk-fill composite resin systems and a conventional incremental composite resin.

## Materials and Methods:

## Study design and tooth selection:

This *in vitro* comparative experimental study was conducted at the biomechanical testing laboratory following institutional ethical approval for the use of extracted human teeth. Eighty freshly extracted intact human maxillary first premolars, obtained from patients aged 18 to 35 years undergoing orthodontic extractions, were collected and stored in 0.5% chloramine-T solution at 4°C for no longer than two months prior to use.

## Inclusion criteria:

Intact maxillary first premolars with fully formed apices, similar crown dimensions (buccolingual width 8.5-10.5 mm, mesiodistal width 6.5-8.0 mm, crown height 7.5-9.0 mm), absence of caries, cracks, or developmental defects.

## Exclusion criteria:

Teeth with visible fracture lines, carious lesions, restorations, hypoplastic enamel, root dilacerations or evidence of resorption. All teeth were examined under a stereomicroscope at 10x magnification to exclude specimens with hairline cracks.

## Specimen mounting:

Each tooth was mounted in a cylindrical stainless steel mold (25 mm diameter x 30 mm height) using auto-polymerizing acrylic resin (Meliodent, Kulzer GmbH, Hanau, Germany). The teeth were embedded up to 2 mm below the cementoenamel junction, simulating the alveolar bone level. A 0.2-0.3 mm layer of polyvinylsiloxane impression material (Express XT, 3M, St. Paul, MN, USA) was applied to the root surface before embedding to simulate the periodontal ligament.

## Endodontic treatment:

Standard access cavities were prepared using an Endo-Z bur (Dentsply Sirona, Charlotte, NC, USA) with high-speed handpiece and water cooling. Working length was established 1 mm short of the radiographic apex. Root canals were instrumented using rotary nickel-titanium files (ProTaper Gold, Dentsply Sirona) to size F2 for the buccal canal and F3 for the palatal canal, following the manufacturer's recommended sequence. Irrigation was performed with 2.5% sodium hypochlorite solution between each file and a final rinse of 17% EDTA for 1 minute followed by distilled water. Canals were dried with sterile paper points and obturated using the continuous wave of condensation technique with gutta-percha (Calamus Dual, Dentsply Sirona) and an epoxy resin-based sealer (AH Plus, Dentsply Sirona). The coronal 3 mm of gutta- percha was removed from each canal orifice and a 2-mm layer of resin-modified glass ionomer cement (Vitrebond Plus, 3M) was placed as a base over the canal orifices.

## Cavity preparation:

Standardized mesio-occluso-distal (MOD) cavities were prepared in all specimens using a high-speed handpiece with diamond burs (ISO #835, medium grit, Komet, Lemgo, Germany) under copious water cooling. The following standardized dimensions were maintained: Isthmus width of one-third the intercuspal distance, pulpal floor depth of 4 mm from the occlusal surface, proximal box height extending to 1.5 mm above the cementoenamel junction and gingival seat width of 1.5 mm. All cavity walls had a 6-degree occlusal divergence and internal line angles were rounded. Remaining buccal and palatal wall thicknesses were maintained at approximately 2.5 mm each. Diamond burs were replaced after every five preparations to ensure consistent cutting efficiency. All preparations were verified using a periodontal probe and digital calipers.

## Group allocation and restoration:

Prepared specimens were randomly allocated into four groups of 20 each using a computer-generated randomization table:

[1] Group 1 (Filtek One Bulk Fill, n=20): 3M, St. Paul, MN, USA

[2] Group 2 (Tetric EvoCeram Bulk Fill, n=20): Ivoclar Vivadent, Schaan, Liechtenstein

[3] Group 3 (SonicFill 3, n=20): Kerr Corporation, Orange, CA, USA

[4] Group 4 (Filtek Z250, Control, n=20): 3M, St. Paul, MN, USA

## Adhesive and restorative procedures:

All cavities were etched with 37% phosphoric acid gel (Scotchbond Etchant, 3M) for 15 seconds on dentin and 30 seconds on enamel, rinsed for 15 seconds and gently blot-dried to maintain moist dentin. A universal adhesive system (Single Bond Universal, 3M) was applied in two consecutive coats with 20 seconds of active rubbing each, gently air-dried for 5 seconds and light-cured for 20 seconds using an LED curing unit (Elipar DeepCure-S, 3M) with an irradiance output of 1,470 mW/cm^2^, verified before each session with a radiometer.

[1] Group 1: Filtek One Bulk Fill composite (shade A2) was placed in a single 4-mm increment and light-cured for 40 seconds from the occlusal surface. A final 2-mm occlusal increment was placed, sculpted to anatomical form and light-cured for 20 seconds.

[2] Group 2: Tetric EvoCeram Bulk Fill (shade IVA) was placed in a single 4-mm increment and light-cured for 20 seconds. The remaining occlusal portion was restored with an additional increment and cured for 20 seconds.

[3] Group 3: SonicFill 3 composite (shade A2) was dispensed using the SonicFill handpiece (KaVo Kerr) at intensity setting 3, which sonically activates the material to reduce its viscosity during placement. A single 5-mm increment was placed, adapted to cavity walls and light-cured for 40 seconds. An additional occlusal increment was placed as needed and cured.

[4] Group 4: Filtek Z250 composite (shade A2) was placed using the conventional incremental technique in oblique layers of 2 mm maximum thickness.

Each increment was light-cured for 20 seconds. Approximately three to four increments were required to complete each restoration. All restorations were finished and polished using aluminum oxide finishing discs (Sof-Lex, 3M) in a sequential coarse-to-superfine protocol. Specimens were stored in distilled water at 37°C for seven days before testing.

## Fracture resistance testing:

Fracture resistance testing was performed using a universal testing machine (Instron 5965, Instron Corporation, Norwood, MA, USA). Each specimen was positioned in the machine so that a stainless steel sphere (6 mm diameter) contacted both buccal and palatal cusps simultaneously, simulating the opposing cusp contact. A thin tin foil sheet (0.5 mm) was placed between the sphere and the tooth surface to ensure even load distribution and prevent stress concentration. A compressive load was applied along the long axis of the tooth at a crosshead speed of 1 mm/min until fracture occurred. The maximum load at failure was recorded in Newtons (N).

## Fracture pattern analysis:

After fracture, all specimens were examined visually and under a stereomicroscope (SZX16, Olympus, Tokyo, Japan) at 10x magnification to determine the fracture pattern. Fractures were classified according to the following categories:

[1] Type I: Fracture of the restoration only (cohesive failure within composite)

[2] Type II: Fracture of the restoration and partial tooth structure above the cementoenamel junction (restorable)

[3] Type III: Fracture of the restoration and tooth structure extending below the cementoenamel junction but not involving the root (potentially restorable)

[4] Type IV: Vertical root fracture or catastrophic fracture rendering the tooth unrestorable

Fractures classified as Types I, II and III were considered favorable (restorable), while Type IV fractures were considered unfavorable (unrestorable).

## Statistical analysis:

All data were analyzed using SPSS version 28.0 (IBM Corp., Armonk, NY, USA). Normality of distribution was assessed by the Shapiro-Wilk test and homogeneity of variances was verified using Levene's test. One-way analysis of variance (ANOVA) was used to compare fracture resistance values among the four groups, followed by Tukey's honestly significant difference (HSD) post-hoc test for multiple pairwise comparisons. Fracture pattern distributions were compared using the chi-square test. The significance level was set at α = 0.05.

## Results:

Fracture resistance values differed significantly among the four restorative material groups (p < 0.001). SonicFill 3 demonstrated the highest mean fracture resistance, followed by Filtek One Bulk Fill and Tetric EvoCeram Bulk Fill, while the conventional composite showed the lowest values. Post hoc comparisons indicated that SonicFill 3 exhibited significantly greater fracture resistance than Tetric EvoCeram Bulk Fill and the conventional composite. Filtek One Bulk Fill also showed significantly higher values compared to the conventional composite. However, no significant difference was observed between Filtek One Bulk Fill and Tetric EvoCeram Bulk Fill, or between Filtek One Bulk Fill and SonicFill 3 (Table 1 - see PDF). The fracture pattern analysis revealed distinct distribution differences among the groups. The majority of fractures in the bulk fill composite groups were favorable (Types I-III), whereas the control group exhibited a higher proportion of unfavorable catastrophic fractures (Type IV). The chi-square test demonstrated a statistically significant association between the restorative material group and fracture pattern classification (χ^2^ = 14.82, p = 0.022). The fracture pattern distributions are presented in Table 2 (see PDF). When fracture outcomes were dichotomized into favorable (restorable) and unfavorable (unrestorable) categories, the bulk fill composite groups collectively demonstrated a significantly higher proportion of favorable fractures compared to the conventional composite group. The combined favorable fracture rate for all bulk fill groups was 85.0% versus 65.0% for the control group. The comparison is detailed in Table 3 (see PDF).

## Discussion:

The results of this study proved that endodontically treated premolars that were restored using bulk-fill composite resins had a much higher fracture resistance than their counterparts that were restored using incremental composite resin. The highest values of fracture resistance were obtained with SonicFill 3 among the bulk-fill materials, but only statistically significant compared with the Tetric EvoCeram Bulk-Fill and conventional composite group. It was based on the null hypothesis that was rejected partially. The high performance of the bulk-fill composite as opposed to the conventional incremental composites can be ascribed to various material based and method based reasons. When a composite restoration is placed in fewer and thicker increments, less interlayer interfaces are present in the restoration body. Any interface of successive increments is a possible plane of vulnerability in which there can be voids, contamination, or incomplete bonding [18]. By reducing these interfaces, a less monolithic and more mechanically homogeneous restoration is produced that can more effectively resist, as well as distribute, the occlusal loading forces [19]. Mechanical advantage of the bulk-fill composites is also associated with the polymerization dynamics of these composites. The composites are developed using modified monomer systems and advanced photoinitiator packages enabling sufficient depth of cure down to thickness of 4 to 5 mm and at the same time providing reduced polymerization shrinkage stress than that of conventional composites [20]. The reduced stress of shrinkage is converted to the minimization of cuspal deflection and enhanced maintenance of the adhesive interface integrity that is especially essential in endodontically-treated teeth whereby the remaining thin dentinal walls stand at risk of fracture due to deformation [21]. The observation that the SonicFill 3 was found to exhibit the highest fracture resistance of all the materials tested is interesting and can be attributed to its special mechanism of placement. The sonic- activation technology in this system minimizes the viscosity of the composite during dispensing process, which enhances the ability to better adapt to the cavity walls and also minimizes the chances of incorporating the voids [22]. When the sonic energy is stopped, the material will come back to a more viscous state and be sculptable and resistant to slumping. This two-step rheology guarantees a close contact of the restorative stuff with the cavity surfaces, maximizing the load- transfer system between the restoration and the remaining tooth structure [23]. The filler composition as well as loading of SonicFill 3 could be another factor that contributes to its high performance. Its filler particles are about 81.3 by weight and this is more than several other bulk-fill composites. An increase in filler loading is usually associated with an increase in the mechanical property such as increased flexural strength, elastic modulus and fracture toughness [24]. It has been postulated that the possibility of a restorative material to show an elastic modulus that is comparable to that of dentin is an essential factor in the even distribution of stresses throughout the tooth-restoration complex to lower the stress concentration at the tooth-restoration interface [25]. Filtek One Bulk-Fill also showed acceptable results, since values of fracture resistance were statistically equal to SonicFill 3. The material is a combination of aromatic urethane dimethacrylate (AUDMA), addition- fragmentation monomer (AFM) and 1, 12- dodecane dimethacrylate monomers which are specific to alleviate polymerization stress by stress-relieving chemistry [26]. The AFM technology enables regulated fragmentation and recombination of polymer chains during polymerization, which is successful in eliminating the accumulated stress in the material and at the bond interface [27]. Though showing higher values of fracture resistance than the conventional composite, Tetric EvoCeram Bulk-Fill was statistically worse than SonicFill 3. This content involves Ivocerin, which is a new germanium-based photoinitiator that improves light reactivity and depth of cure. It uses also a pre-polymerized filler techno and stress relieving monomers [28]. Nonetheless, the reduced weight content of filler relative to SonicFill 3 and the variation in the morphology of the filler could explain the slightly lower values of fracture resistance measured [29]. Fracture pattern analysis gives information of clinical value in addition to the actual values of fracture resistance. The much higher number of favorable, restoreable fractures in the bulk-fill composite groups (85% combined) than the conventional composite group (65) seems to indicate that bulk fill composites do not only strengthen the remaining tooth structure but also have a modifying effect on the fracture behavior, which is more favorable. This observation is similar to past documents that show that restorative materials with the right elastic moduli and high adhesive integration favor fracture patterns that are still above the bone level and hence secondary treatment becomes possible [30]. The fact that the percentage of Type IV catastrophic fractures is relatively high in the conventional composite group (35%) is a worrying factor. Root fractures that are catastrophic require extraction and clinical outcomes are worst possible in a tooth that has undergone endodontic restoration. The increased rates of these fractures in the incremental composite group are possibly due to composite polymerization stresses due to multiplicity of bonded increments that can generate intricate internal stress patterns deviation of crack propagation in less desirable pathways through the root [31]. It is necessary to mention that the average values of fracture resistance obtained by all groups in this research were higher than the maximum values of occlusal forces reported to exist in the premolar teeth (ranging between 200 and 450 N) when these teeth are used in normal masticatory activities. The minimum mean value seen (968.4 N in control group) is significantly greater than the physiological loading conditions [32]. Nevertheless, teeth within the oral cavity experience cyclic fatigue load during prolonged durations and the measured values of fracture resistance in the laboratory which are static could be inflated relative to the clinical fracture threshold. It is generally assumed that higher levels of material provide more safety margins against fatigue induced failure [33]. Simulation of periodontal ligament by the thin layer of elastomeric material is a methodological improvement in which the clinical relevance of fracture testing is enhanced. In the absence of PDL simulation, the stresses can be concentrated in acrylic-tooth interface, resulting in artificially low fracture values and the analysis of unrealistic fracture patterns [34]. It was decided to use a 6-mm steel sphere with contact between the two cusps at the same time to provide the worst-case occlusal loading condition since clinical cusp fractures often happen under the influence of a hard food bolus in contact with more than one cuspal slope [35]. There are a number of weaknesses that should be considered when interpreting these findings. *in vitro* design is not capable of recreating the highly complex biomechanical conditions of the oral cavity, such as cyclic loading, exposure to moisture, changes in temperature and enzymatic degradation. The fatigue mechanism, which is considered to be the primary mode of failure of the restored teeth in the clinic, is not considered, despite the similarity of data in the comparative fracture resistance of the use of a static loading protocol. The artificial cavity model used to prepare the cavity is standardized, but might not be a full reflectance of the variable clinical presentations that are experienced in practice [36]. The research should be supplemented with the use of fatigue testing, thermomechanical aging and finite element analysis in the future to present data on performance of these materials in more clinically relevant environments [37].

## Conclusion:

Bulk-fill composite resins enhance the fracture resistance of endodontically treated premolars compared to conventional restorative approaches. Material properties and placement techniques play a key role in determining both strength and fracture patterns. Appropriate selection of restorative materials can improve durability and clinical success of endodontically treated teeth.
